# Editorial: Long-term perspectives in advanced heart failure therapies

**DOI:** 10.3389/fcvm.2023.1220000

**Published:** 2023-06-12

**Authors:** Felix Schoenrath, Finn Gustafsson, Claudius Mahr, Ivan Netuka

**Affiliations:** ^1^ Department of Cardiothoracic and Vascular Surgery, Deutsches Herzzentrum der Charité, Berlin, Germany; ^2^ Partner Site Berlin, German Center for Cardiovascular Research (DZHK), Berlin, Germany; ^3^ Charité University Medicine Berlin, Berlin, Germany; ^4^ Department of Cardiology, Rigshospitalet, University of Copenhagen, Copenhagen, Denmark; ^5^ Division of Cardiology, Department of Medicine, School of Medicine, University of Washington, Seattle, WA, United States; ^6^ Department of Cardiovascular Surgery, Institute for Clinical and Experimental Medicine (IKEM), Prague, Czechia

**Keywords:** advanced heart failure (AHF), LVAD (left ventricular assist device), heart transplantation (HTx), short term mcs, medical therapy, heart failure

**Editorial on the Research Topic** Long-term perspectives in advanced heart failure therapies

Advanced heart failure is increasingly prevalent and the most resource-consuming entity of the global heart failure pandemic.

In addition to established treatment options like cardiac transplantation and long-term mechanical circulatory support, other contemporary approaches, such as short-term mechanical circulatory support and medical therapies expand the therapeutic armamentarium.

This research collection addresses these aspects, as well as current developments in the diagnostics of advanced heart failure and treatment optimization after cardiac transplantation or LVAD implantation in terms of early postoperative, as well as long-term outcomes.

The article by Keyt et al. reviews the unique pathophysiology of thin filament mutations, an important recently-discovered etiology of cardiomyopathies leading to cardiomyopathy. They further emphasize the relevance of personalized treatment options and give an overview of current therapies for this specific scenario.

In regards to medical therapies in advanced heart failure, the selected articles focus particularly on the role of the phosphodiesterase-5 inhibiting drug sildenafil, and the calcium sensitizer and potassium channel opener levosimendan.

Abdelshafy et al. systemically review the current evidence of levosimendan in the treatment of advanced heart failure, especially in the perioperative LVAD setting to prevent or mitigate postoperative right heart failure. They found advantageous hemodynamic conditions, which so far did not translate into clinical benefits; the stage is set for a well-conducted prospective randomized clinical trial in this setting in the future.

Monzo et al. investigated clinical variables that could be associated with the hemodynamic response to sildenafil in a subset of patients with pulmonary hypertension fulfilling criteria for vasoreactivity testing. They identified serum potassium levels, serum aldosterone levels and atrioventricular valve regurgitation as potential cofounders of hemodynamic response to acute administration of PDE5i.

With temporary mechanical circulatory support becoming a more prevalent option as a bridge to recovery, or to durable advanced surgical therapies, potential complications and problems inherent to intermediate-term support warrant further investigation. Sugimura et al. evaluated the outcome of patients supported by microaxial flow pump systems after re-implantation procedures. Re-implantation was required in approximately one in seven patients in their single center cohort, and could be performed safely. Importantly, half of all system exchanges were required due to pump thrombosis. These results merit further analysis of anticoagulation strategies.

Comprehensive and forward-thinking articles are collected exploring the latest advances in LVAD therapy.

Maw et al. were able to demonstrate the safety, short-term efficacy and physiological responsiveness of a sensorless automated speed control system for a centrifugal LVAD in a pilot study. They were able to increase support during physical exertion and decrease support during resting times. With this, an important first step towards “smart pumps” and therefore improved hemocompatibility (less suction events, as well as better quality of life (better exercise tolerance) has been made.

Kortekaas et al. evaluated the treatment success of systemic thrombolysis with a structured protocol as an alternative treatment strategy to pump exchange in patients with VAD thrombosis. They showed that a systemic thrombolysis is feasible and successful in the majority of cases and might be a reasonable treatment alternative, especially in patients at high surgical risk, and in those who would require a complete system exchange (e.g., HVAD to HeartMate 3).

Nozdrzykowski et al. compared the standard sternotomy surgical approach to LVAD implantation with less invasive surgery with respect to morbidity and mortality during the first postoperative year in a large contemporary LVAD cohort. They were able to demonstrate potential advantages of less invasive surgical procedures during the very early postoperative period.

Finally, four articles deal with relevant topics in cardiac transplantation medicine.

Starting with Rivinius et al., they carefully investigated whether preoperative type II diabetes mellitus in cardiac transplant recipients has an impact on postoperative outcome. They were not only able to present acute postoperative results, but also stratified outcomes up to five years post-transplant analyzing the impact of postoperative HbA1c control. Thus, they highlight the importance of optimal glycemic control after cardiac transplantation.

Kooij et al. summarize their institutional experience regarding the important topic of sinus node- and conduction-system disturbances during the early post-transplant period. In their analysis, theophylline remains a valuable alternative to permanent pacemaker implantation and might be able to facilitate weaning of chronotropic support.

Further insights into the early postoperative period after heart transplantation are gained with the findings from Zhao et al. They evaluated the impact of extracorporeal membrane oxygenation on right ventricular function after heart transplantation. Interestingly, recipients preoperative right ventricular characteristics most likely display a certain degree of pulmonary vasculature damage that has relevant influence on the postoperative need of ECMO and therefore a significant impact on short- and long-term survival after transplantation.

McDonald et al. studied the histopathological phenomenon of acute myocardial injury (AMI) after cardiac transplantation. AMI is a further finding in endomyocardial biopsies, scientifically underrepresented when compared with grading of humoral or cellular rejection. They were able to correlate these findings with primary graft dysfunction and reduced survival. The authors suggest ischemia and myocardial reperfusion injury as primary contributors to those structural changes.

Overall, this research collection strives to provide a comprehensive and forward-thinking perspective on some of the latest advances in advanced heart failure diagnosis and therapy, paving a path forward for improved patient outcomes and better quality of life. It emphasizes an urgent need for precision medicine and the expansion of our therapeutic options beyond the conventional approach.

